# Corn Starch (*Zea mays*) Biopolymer Plastic Reaction in Combination with Sorbitol and Glycerol

**DOI:** 10.3390/polym13020242

**Published:** 2021-01-12

**Authors:** M.D. Hazrol, S.M. Sapuan, E.S. Zainudin, M.Y.M. Zuhri, N.I. Abdul Wahab

**Affiliations:** 1 Advanced Engineering Materials and Composites Research Centre, Department of Mechanical and Manufacturing Engineering, Universiti Putra Malaysia, Serdang 43400, Selangor, Malaysia; hazrolpostgrad@gmail.com (M.D.H.); edisyam@upm.edu.my (E.S.Z.); zuhri@upm.edu.my (M.Y.M.Z.); 2 Laboratory of Biocomposite Technology, Institute of Tropical Forestry and Forest Products (INTROP), Universiti Putra Malaysia, Serdang 43400, Selangor, Malaysia; 3 Advanced Lightning Power and Energy Research (ALPER), Department of Electrical and Electronic Engineering, Universiti Putra Malaysia, Serdang 43400, Selangor, Malaysia; izzri@upm.edu.my

**Keywords:** corn, starch, sorbitol, glycerol, plasticizer, biodegradable films

## Abstract

The research included corn starch (CS) films using sorbitol (S), glycerol (G), and their combination (SG) as plasticizers at 30, 45, and 60 wt %, with a traditional solution casting technique. The introduction of plasticizer to CS film-forming solutions led to solving the fragility and brittleness of CS films. The increased concentration of plasticizers contributed to an improvement in film thickness, weight, and humidity. Conversely, plasticized films reduced their density and water absorption, with increasing plasticizer concentrations. The increase in the amount of the plasticizer from 30 to 60% showed a lower impact on the moisture content and water absorption of S-plasticized films. The S30-plasticized films also showed outstanding mechanical properties with 13.62 MPa and 495.97 MPa, for tensile stress and tensile modulus, respectively. Glycerol and-sorbitol/glycerol plasticizer (G and SG) films showed higher moisture content and water absorption relative to S-plasticized films. This study has shown that the amount and type of plasticizers significantly affect the appearances, physical, morphological, and mechanical properties of the corn starch biopolymer plastic.

## 1. Introduction

Plastic waste and pollution are globally ubiquitous and found throughout the ocean, lakes, rivers, in soils and sediments, in animal biomass, and the atmosphere [1,2,3]. Present environmental issues, as environmental obstacles, such as non-biodegradable waste products, plant waste, and rising waste mountains are progressively reported [4,5,6]. The landfill areas are limited, and expanded incineration capacity requires high capital investment and intensifying environmental risks. These problems have contributed to the design and development of the environmentally sustainable and renewable materials as substitutes to the traditional non-biodegradable materials [7,8,9].

The reduction in dependency upon plastic and petroleum products is one justification for the study of biomass in polymer composite applications [10,11,12]. Moreover, the use of biomass such as kenaf [13], sugar palm [14,15], water hyacinth [16], ginger [4,17], and sugarcane bagasse [18,19] to strengthen the composites might contribute to partial waste degradation, which in turn help to solve environmental problems [15,20,21]. The widespread use of biopolymers in the place of standard plastics would help to reduce the weight of waste. Therefore, biodegradable materials take part in the natural cycle “from nature to nature” and play an important role for environmental sustainability [22]. Natural polymers based composites offers significant advantages over synthetic fibre reinforced petroleum matrix based composites with regard to biodegradability, biocompatibility, design flexibility, and sustainability [23]. 

Biorenewable materials have been extensively used as a matrix, miscible agent, or reinforcement in many applications [24]. In the development of innovative methods and materials, composites offer important advantages because of their excellent properties such as ease of fabrication, higher mechanical properties, high thermal stability, and many more [25]. Along with the properties of starch, plasticizers properties also have an important role in the morphology and properties of the resulting polymers. The efficiency of polymer is further improved strongly by the plasticizer for mechanical, thermal, and electrical properties [26,27].

Corn is amongst the most plentiful sources of plant residues, providing various benefits, including high starch levels, excellent consistency, performance, ease availability, and biodegradability [4]. According to Sanyang et al., [2], corn is one of the world’s most widely available agricultural cereals. There are usually six corn types: dent, flint, pop, pod, flour, and sweet corn. The physical–chemical properties of these kinds differ considerably due to environmental effects [5]. According to Attache Reports USDA, corn production in 2020 was estimated at 4.04 billion tons, representing 31% of global cereal production, making maize the third-largest food grain. The corn plant consists of one or more stems, which are connected with a short root combination. The branch ends with inflorescences at each node. After harvest, the plant parts are converted into disposable waste [6].

Corn is a critical source of major starch, a kind of alpha-linked glucose that is commonly used as a gelling, water retention, and bulking agent in food factories [7]. Most of the corn kernel composition was starch, with the balance being sugar, protein, oil, and ash. Many natural fibers, including stalk, stalk, straw, leaf, and husk, can be extracted from this plant. Compared to other agricultural bioproducts, corn fibers deliver different characteristics, such as a 90% saving and being more available than other natural fibers [8]. Cornstarch is a semi-crystalline biopolymer that is comprised a blend of linear polysaccharide amylose (20–28%) and highly unitary amylopectin [28]. Several studies in the development of biopolymer films with different plasticizing agents using thermoplastic corn starch were conducted [29,30]. Sun et al. [31] produced TPS film using cornstarch and urea plasticizer. The result shows that the mechanical properties of the starch biopolymers increase with the increase of urea plasticizer. Starch-based materials have low water–barrier characteristics and poor mechanical efficiencies compared to synthetic polymers due to their high hydrophilicity and water affinity [16,32,33,34,35].

Plasticizers are relatively non-volatile organic (mainly liquids) compounds. When integrated into a plastic or elastomer, they increase the versatility, extension, toughness, and processability of the polymer [36]. Plasticizers are among the plastic industry’s most regularly used additives. They are typically cheaper than other polymer additives. Plasticizers in PVC, the third-largest polymer by volume after PP and PE, are most widely used. PVC is, in turn, used in a number of items. Various plasticizers, however, are used in starch, e.g., sugar palm sugar [37], corn [36], and cassava [38], to improve the mechanical strength and thermal properties of biofilms. Plasticizing is the most popular method because the low-cost liquid plasticizers allow the formulator freedom to formulate a variety of formulations (from semi-rigid to highly flexible, depending on quantity) [3]. Plasticizers are most commonly used in esters formed from alcohol reactions of acids or acid anhydrides. Thus, the benefits of using the plasticizer include improved elongation, softness, solvency, lubricity in the surface, decreased viscosity, improved thermal stability, and flexibility [6].

In 1872 the French chemist Joseph Boussingault first found sorbitol in the berries of mountain ash. Sorbitol is a common source of dietary in many plants such as apples, pears, peaches, prunes, and berries. Chemical products may also be produced to be used as a sweetener in some foods and beverages such as candies, cookies, pudding, and oatmeal. It is also produced commercially from corn syrup for use in packaged foods, beverages, and medicines [24]. Glycerol is the most essential triol present in natural fats and oils as fatty esters. It is mostly regarded as a byproduct of soap production after 2800 BC. Glycerol currently has a wide variety of uses in the personal care industry, dairy, polyol, resin, tobacco, detergent, and pharmaceutical industries [39].

This study, therefore, mainly aims to construct and analyze corn starch-based biopolymer films and plasticizer. This work also intends to assess the potential utilization of the highly abundant and costly disposal of corn plants residues, which can help alleviate waste problems, respond to community demand for agricultural and polymer waste disposal, and boost economic development through transfers from waste to wealth [20]. Corn starch with plasticizer adds value to waste goods and increases the eco-friendly suitability of the starch-based biopolymer. It should be noted that corn starch particles used in the present study were not chemically treated or thermally modified and were filtered using particle size analyzer before processing into biopolymer plastic.

Thus, this study focuses on investigating the potential effects of using glycerol, sorbitol, and a combination of glycerol and sorbitol as plasticizers on the physical, mechanical, water barrier, and morphology properties of the corn starch biopolymer at varying concentration.

## 2. Methodology

### 2.1. Materials

The commercialized Star brand corn starch was collected from a local factory, Thye Huat Chan Sdn. Bhd., Sungai Buloh, Selangor, Malaysia. The glycerol and sorbitol plasticizers were supplied by Evergreen Engineering & Resources Sdn Bhd, Semenyih, Selangor, Malaysia. The starch was characterized in powder basis and grading in sieve machine Matest A060-01, MATEST S.p.A, Ancore, Italy to 0.25 mm in size.

### 2.2. Corn Starch Biopolymer Preparation

**Figure 1:**
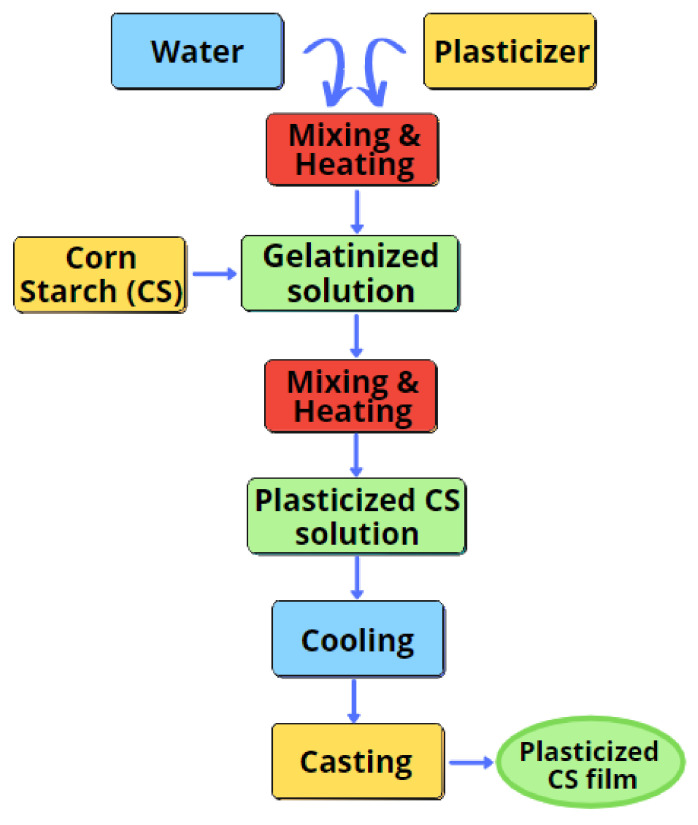
Flow chart of film preparation.

The cornstarch-based films were manufactured by conventional solution casting procedures, as shown in Figure 1. In total, two types of plasticizers were added into a beaker containing 180 mL of distilled water. The beaker was placed into the water bath at a temperature of 85 ± 5 °C for 20 min. This phase is crucial to produce a homogeneous suspension. Then, a 10 g of pure corn starch was individually added into the solution at concentrations 0, 30, 45, or 60% (*w*/*w*, aqueous dispersion of gelatinized corn starch). Solution heating continued at the same temperature for another 20 min. Before casting on a thermal platform, the slurry was let to cool. The casting dishes were then weighed at 45 g to maintain uniformity on film thickness. The blend was then dehydrated at 65 °C for 15 h in an air circulation oven. The films were dehydrated from the casting plates and kept at room temperature for one week in plastic bags before characterization. Films formed by plasticizer category and concentration were encoded as follows: G30%, G45%, G60% for glycerol, S30%, S45%, S60% for sorbitol, SG30%, SG45%, and SG60% for sorbitol/glycerol, and CS for non-plasticized corn starch film (control).

### 2.3. Particle Size Distribution (PSD)

Using an integrated Q-space feeder, a mastersizer 2000 E Ver. 5.52 (Malvern Instruments Ltd, Worcestershire, UK) was used to classify the particle size distribution of powdered corn samples. PSD measures dry-based dispersed particle proportions over specified particle size ranges, essential to control the efficiency of stress transmission between molecules. Particle size analysis is a rather standard indicator used in many different industries for quality control. Particle size is a crucial factor in assessing production efficiency and end product consistency over almost every industry that involves milling or grinding. Before the distribution analysis, the particle size of the measured samples was examined with a 1000 μm sieve.

### 2.4. Film Thickness

Film thickness was measured using a 0.001 mm sensitive digital micrometer (Mitutoyo Co., Kanagawa, Japan) collected for each sample from five different film areas. The average value of measurements was used for each sample.

### 2.5. Film Density

A densimeter (Mettler-Toledo (M) Sdn. Bhd., Selangor, Malaysia) was used to assess the density of the produced films. The dipping solvent used in this work was xylene instead of distilled water to prevent hydrophilic film samples from taking water. Furthermore, the solvent density must be less than the film to ensure that the film does not float. Xylene is also much more desirable than water because of its low density. The samples were dried in desiccators for 7 days, with SiO_2_ as the drying agent. The initial dry matter of each plasticized film was then measured. Until immersing the film sample in the liquid, it was weighted (*m*). The amount of liquid displaced by the film was reported as *v*. Equation (1) was used for the density measurement (*ρ*). The test was conducted in three replicates.

*ρ* = *m*/*v* (1)

### 2.6. Film Moisture Content

A digital weighing scale was used to measure the initial weight of the film samples (*W_i_*). After drying in a 105 °C oven for 24 h, the samples were weighed (*W_f_*). The moisture content of each film sample was calculated using Equation (2). The triple test was carried out and the moisture content was recorded as the mean for each film.

*Moisture content* = (*W_i_* − *W_f_*)/*W_i_* × 100 (2)

### 2.7. Water Absorption (WA) 

A 15 mm^2^ film sample was dried in a laboratory oven at 105 °C for 3 h, then cooled and immediately weighed (*M_i_*). The selected sample was immersed in 100 mL of distilled water at room temperature. The sample was taken out of the water after a particular immersion time, wiped with a smooth cloth, and reweighed (*M_f_*). A total of three test replicates were performed and the mass differences between the initial and the immersed films were used to evaluate the *WA* using Equation (3) below:

*WA* (%) = (*M_f_* − *M_i_*)/*M_i_* × 100 (3)

### 2.8. Fourier Transform Infrared Spectroscopy (FTIR)

The presence of functional groups was detected by an infrared spectrometer model (Bruker vector 22, Lancashire, UK). During the test, the FTIR spectrum of 4000 to 400 cm^−1^ with a spectral resolution of 4 cm^−1^ was measured. The samples were mixed with KBr, after which the mixture was pressed into thin transparent films that were analyzed.

### 2.9. X-Ray Diffraction (XRD)

A Shimadzu LabX XRD 6000 (Shimadzu Corporation, Kyoto, Japan) was used for the XRD test. The system measurements were performed with the new software suite X’Pert HighScore Plus, an integrated software platform that incorporates both measurement and analysis functions. The test was conducted at a scattering angle speed of 1° (θ) min^−1^ within angular values from 5° to 60° (2θ). The tube voltage and current were defined at 40 kV and 35 mA. The outcomes from XRD test include relative crystallinity (*X_c_*), crystalline area (*I_c_*), and amorphous area (*I_o_*). Equation (4) defines relative crystallinity as a ratio between crystalline and amorphous space.

*X_c_* (%) = (*I_c_* − *I_o_*)/*I_c_* × 100 (4)

### 2.10. Tensile Properties of Films

A universal tensile machine (5kN INSTRON, Instron, Norwood, United States) measured the mechanical behavior of the specimens. The test was conducted according to D882 (ASTM, 2002). The tensile machine clamps were attached to a film strip (70 mm × 10 mm) that was pulled at 2 mm/min crosshead speed, with an effective grip distance of 30 mm. The tensile machine was linked to computing software, Bluehill 3, which used a mean calculation of five replicates for each specimen to assess the results of tensile power, elastic modulus, and elongation at the breakpoint.

### 2.11. Scanning Electron Microscopy (SEM)

The analysis of surface morphology of the samples was performed using a scan electron microscope (COXEM EM-30N, Coxem, Daejeon, Korea). Each sample was immersed in nitrogen liquid and coated with a thin layer of gold (0.01–0.1 μm) and placed on a bronze grid prior to examination. The scans were conducted with liquid nitrogen to freeze the films in a high vacuum condition. The electron beam was then emitted by a 20 kV voltage that communicated with the sampling atoms to produce signals that contain information on superficial topography and produce high-resolution images at different magnification factors.

### 2.12. Statistical Analysis

SPSS software was used to perform the analysis of variance (ANOVA) on the obtained experimental results. Duncan’s test was used to conduct means comparisons at a 0.05 level of significanc (*p* ≤ 0.05).

## 3. Results and Discussion

### 3.1. General Appearance Corn Starch (CS) Plasticized Films

**Figure 2:**
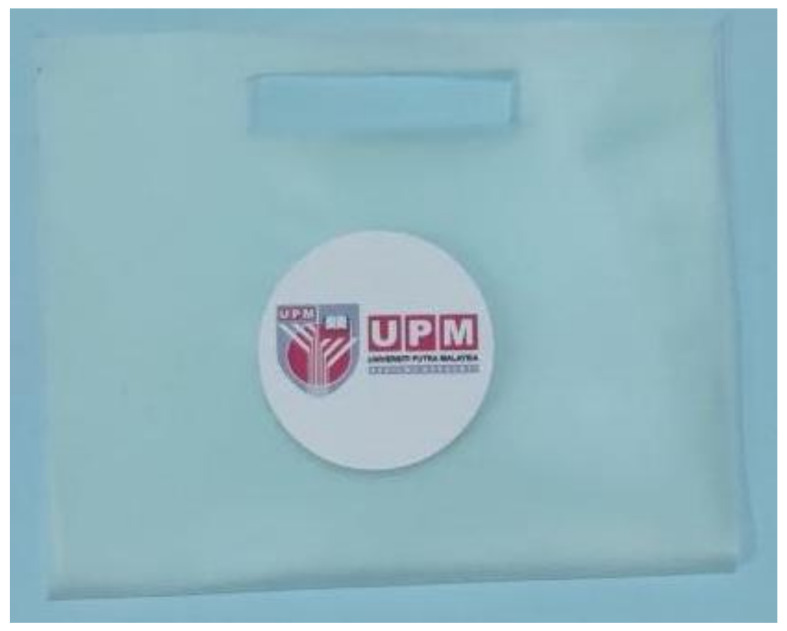
Corn starch (CS) with 30% sorbitol film prepared using the solution casting method.

Figure 2 shows the photographic images of the obtained plasticized CS plastic films, whereas Table 1 describes their visual appearances from non-plasticized to plasticized CS films, annotated as control films. The control corn starch films prepared from zero plasticizer were somehow brittle, rigid, and fragile. Many cracks on the film surface were observed. They broke into bits, and hence, their peeling and handling was difficult. This finding can be due to strong inter/intra molecular hydrogen CS bindings, resulting in brittle and stiff films with surface cracks that gave the macromolecular chains less movement. These observations were in agreement with the findings of Suppakul, Sanyang, and Ilyas et al. [40,41,42], who prepared cassava, potato, and sugar palm starches, respectively. Furthermore, the appearance of all plasticized films is described in Table 1.

### 3.2. Particle Size Distribution (PSD)

**Figure 3:**
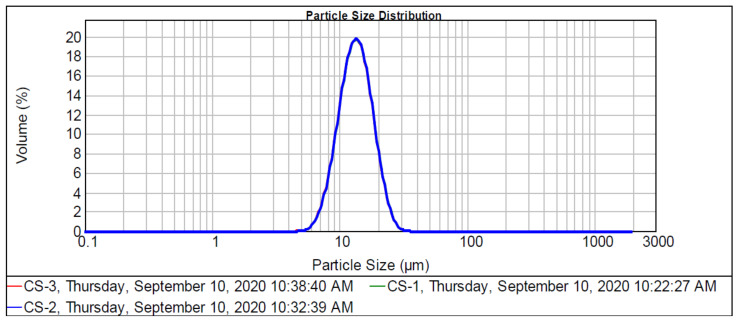
Particle size distribution (PSD) of corn starch.

A polymers strength depends on the efficacy of homogeneous polymeric matrix. Polymers strength is highly influenced by particle size distribution, particle charge, and particle/matrix interfacial strength parameters [43]. The refractive index or the ratio of light speed in a vacuum to the light speed in CS was denoted as 1.3344. This value was taken from Malvern Instrument Mastersizer 2000 sample dispersion and refractive index guide. Thus, the PSD of corn starch is presented in Figure 3. The curve was analyzed to estimate the gradation size range of CS particle used in the current study. The graph revealed that 10% of starch particles had dimensions of less than 10 μm and 50% of the sizes were 10 to 15 μm, respectively. Majority of the corn starch particles, however, had sizes below 20 μm; these findings were similar to the results of [36,44,45].

### 3.3. Film Thickness and Weight

From Table 2, it was found that the thickness of CS-based films showed a proportional increase to the increasing plasticizer percentage concentration, irrespective of plasticizer type. In response to the increase in S, G, and SG concentrations (30 to 60 %), the films’ thickness was increased from 0.16 to 0.22, 0.14 to 0.19 mm, and 0.18 to 0.20 mm, respectively. This observation might be attributed to the plasticizers’ role in the reconstruction and restructuring of intermolecular chain networks that translate to the increase of film thickness. The effect of the plasticizers concentration on film thickness was similar to Ibrahim, Sanyang, Razavi, Jouki, Edhirej et al. [36,41,46,47,48]. Furthermore, various plasticizer forms demonstrated significant impacts on the film thickness, as shown in Table 2. Plasticizing films from S- and SG- showed thicker films than G-plasticized films.

The film thickness variations of different plasticizers might be associated with their molar mass, as the formulation of the film solution was consistent. S30, G30 and SG30-plasticized film showed the lower weight ranging from 0.06 mg to 0.07 mg compared to S60, G60, and SG60-plasticized film ranging from 0.07 mg to 0.09 mg. The low thickness and weight of films with G-plasticized films might be due to lower molar mass in comparison to S-plasticizer. Ghasemlou et al. [49] stated as well that films plasticized by S yield thicker films than films plasticized by G.

### 3.4. Film Density

From Table 2, adding plasticizers reduced the CS density from 1.65 g/cm^3^. Therefore, the plasticized films were less dense than the unplasticized CS film. The effect on the density of CS films of the plasticizer types and concentrations are shown in Table 2. Raising the concentration of plasticizers from 30 to 60% caused slight decrements in the density of S- (1.45–1.43 g/ cm^3^), G- (1.39–1.30 g/cm^3^), and SG-plasticized films (1.40–1.38 g/cm^3^). It can be shown that the film density was marginally decreased, regardless of the plasticizer types by increasing the percentage of plasticizers from 30% to 60%. The results of the plasticized films S, G, and SG were consistent with the results obtained by Sanyang, Sahari et al. [41,50], who used a dry treatment technique (hot pressing) to plasticized sugar palm starch with glycerol (15, 30, and 45%). The density values did not indicate any difference between the different types of plasticizers.

However, the decreasing order of density is the following: S, SG, and G plasticized films of the same plasticizer concentration. The disparity in molecular weight and density of plasticizers might be due to this phenomenon. In addition, Razavi et al. [46] did not find any substantial difference between wise seed gum (SSG) films containing glycerol and sorbitol, although the density of glycerol films plasticized was below 60% lower than that of sorbitol.

### 3.5. Film Moisture Content

For all plasticized CS-films, moisture content was significantly increased as the concentration of plasticizers rose from 30% to 60%; except for S-plasticized films which only showed slight increment. In general, starch films turned out to be hydrophilic with rising levels of plasticizers. Thus, several investigations reported that the moisture content of hydrocolloid films increased by adding more plasticizer [36,41,48,51,52,53,54].

However, as for G- and SG-plasticized films, it was not noticeable how sorbitol affected the humidity of CS films. The moisture content of S-plasticized films with increasing concentration of plasticizers is shown in Table 2. Similar findings were reported by Ibrahim, Sanyang, Edhirej, Sun, Ilyas et al. [36,41,48,51,55]. The low moisture content of S-plasticized films can be explained by the molecular similarity of glucose units to that of sorbitol, as compared with film-containing glycerol (e.g., G- or SG plasticized film) and thus by stronger molecular interactions between the sorbitol and the intermolecular polymer chains. Consequently, the probability of sorbitol interacting with water molecules was smaller.

On the contrary, the hydroxyl groups in glycerol had a strong affinity with water molecules; enabling glycerol containing films to easily retain water within their matrix and form hydrogen bond [56]. Hence, glycerol acted as a water-holding agent, whereas sorbitol acted as water resistant agent. Although, Ibrahim et al. [36] reported stagnant moisture content of CS films (17.28, 18.01, and 17.88%) with increment in sorbitol concentration (25, 45, and 55%, respectively), the moisture content of S-plasticized films (9.25, 9.58, and 10.04% for 30, 45 and 60% sorbitol concentrations, respectively) obtained in this study were generally lower.

### 3.6. Water Absorption (WA)

**Figure 4:**
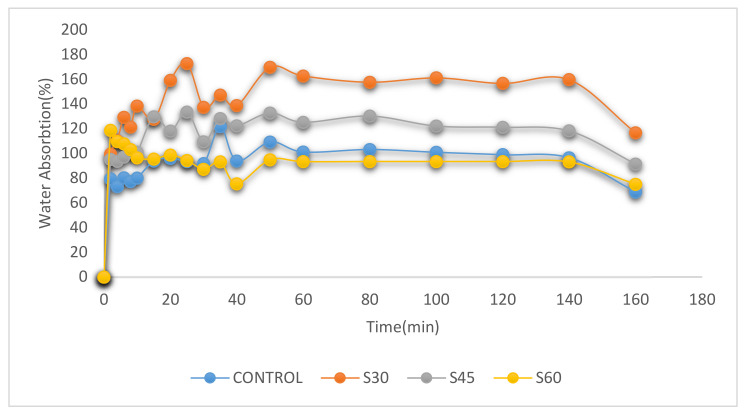
Water absorption on CS films with sorbitol plasticizer.

**Figure 5:**
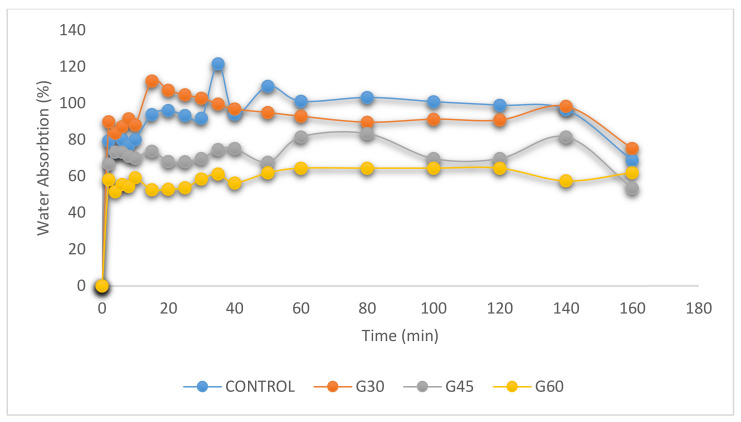
Water absorption on CS films with glycerol plasticizer.

**Figure 6:**
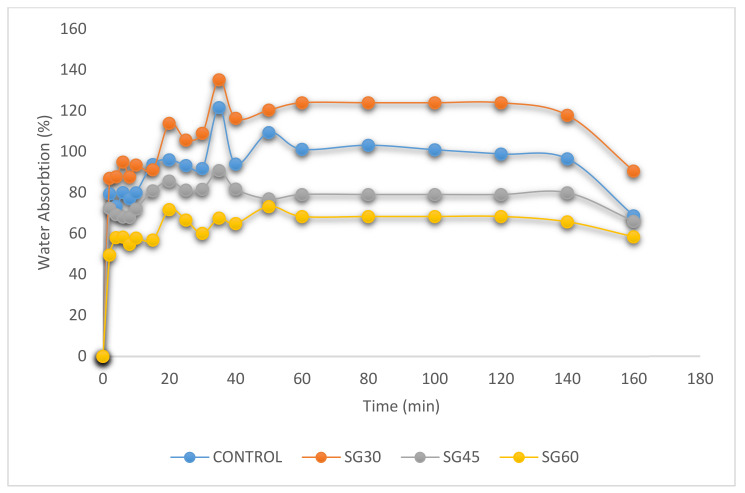
Water absorption on CS films with sorbitol/glycerol plasticizer.

Figure 4, Figure 5 and Figure 6 display the percentage of weight gain based on water intake for the CS-plasticized film. For starch films, water absorption is important because water works as a plasticizer. Plasticized films at higher plasticizer and moisture content had greater flexibility [36,41]. The impact of the immersion time was restricted to 160 min because the film samples started to soluble in water at 140 min high hydrophilicity of plasticized films with greater plasticizer content [57]. Higher tendency of water absorption into plasticized polymers was due to the formation of hydrogen bonds with starch caused by the presence of hydroxyl groups within the plasticizer molecules. For corn starch films with different type of plasticizer and material, the water absorption percentage shows Figure 4, Figure 5 and Figure 6 as functions of the time.

Interestingly, both films absorbed high volume of water at room temperature during the early 20 min of water immersion. The water absorption of the films was improved with higher plasticizer content because of their water-soluble and naturally hygroscopic properties [57,58]. In 40 min, the control film absorbed about 120%, while in S-, G- and SG- plasticized films, the films absorbed approximately 147%, 112%, and 135% of the plasticized films. After 140 min, the control film and all films with different plasticizers types and content began to dissolve in water, except for 60% glycerol-plasticized films.

### 3.7. Fourier Transform Infrared Spectroscopy (FTIR)

**Figure 7:**
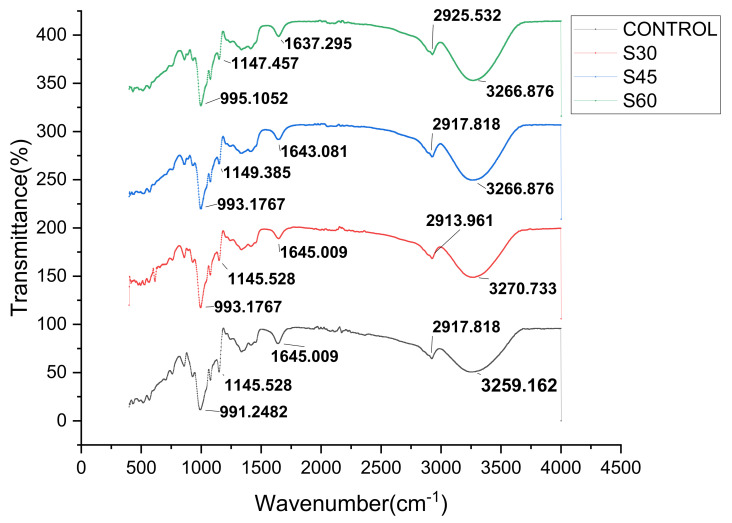
FTIR curves of CS films with various sorbitol concentration.

**Figure 8:**
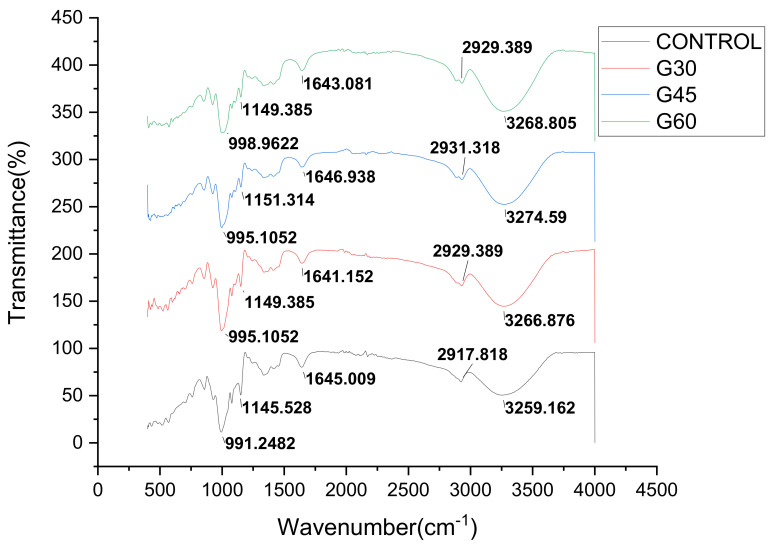
FTIR curves of CS films with various glycerol concentration.

**Figure 9:**
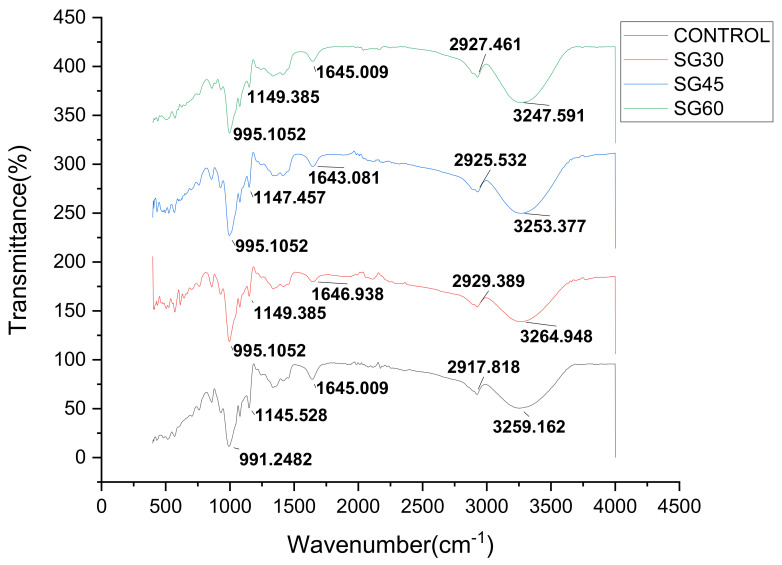
FTIR curves of CS films with various sorbitol/glycerol concentration.

The IR spectrum of unplasticized and plasticized CS films are presented in Figure 7, Figure 8 and Figure 9. Cornstarch chemical structure was given by C_6_H_10_O_5_, while sorbitol and glycerol were C_6_H_14_O_6_ and C_3_H_8_O_3_, respectively. The FTIR technique was employed to determine the variations in the compositional structure of starch and evaluate the potential interactions between plasticizer [59,60,61]. The FTIR spectrum curve was split into four main regions as follows: the first region above 3000 cm^−1^ wavenumbers, the second region is ranged from 2800 to 3000 cm^−1^ wavenumbers, the third wavenumber region from 800 to 1500 cm^−1^, and the last region will cover up 800 cm^−1^ and below. The control film showed peaks at 991.24, 1145.52, 1645.01, 2917.82, and 3259.16 cm^−1^. The peak observed around 3259.16 cm^−1^ was associated with the stretching of the O-H groups at the end of the polymer chain of starch and plasticizer, where the band identified at 2917.82 cm^−1^ was attributed to C–H stretching. The peak 1645.01 cm^−1^ was assigned to the bending mode of the absorbed water O–H and the peak around 1145.52 was assigned to C–O bonding. The peak at 991.25 also showed that C–H molecules started to vibrate, causing H atoms to separate from C.

As seen in Figure 7, Figure 8 and Figure 9, the FTIR spectra curves were similar because the elemental composition of the plasticized films was based on the starch structure [21,62,63]. For the region between 2800 and 3000 cm^−1^, the oscillations of water fragments led to the emergence of the wide infrared band at the peak of 3259.16 cm^−1^ [36,41,48,51]. Similarly, the occurrence of C–H vibrational stretching resulted in a sharp peak at 2917.82 cm^−1^. As a result, it was noticed that the FTIR spectra of S,G, and SG films presented identical spectra peaks irrespective of plasticizer types and concentrations with respect to plasticizers addition. For example, the only difference was that the sharp summit that emerged from the stretching of the O-H group was marginally reduced to lower wavenumbers. The previously described results showed that all films showed absorption levels in the same regions irrespective of the types and concentrations of plasticizers. This indicated that all films possessed identical functional groups, known as polyols [36,41]. In addition, from the FTIR study, applying plasticizers to CS-films was shown to not altering the chemical composition of CS. This showed that the chemical frames of the resulting CS-films were entirely stable and no significant chemical reactions occurred during the addition of plasticizer.

### 3.8. X-Ray Diffraction (XRD)

**Figure 10:**
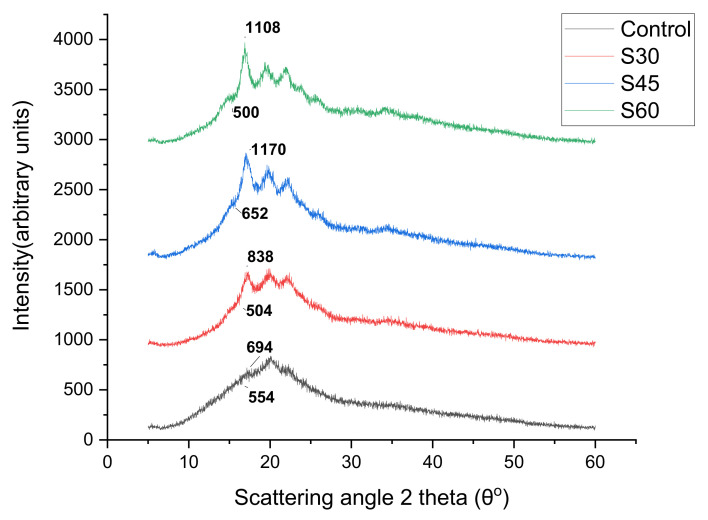
XRD of sorbitol plasticized and unplasticized CS films.

**Figure 11:**
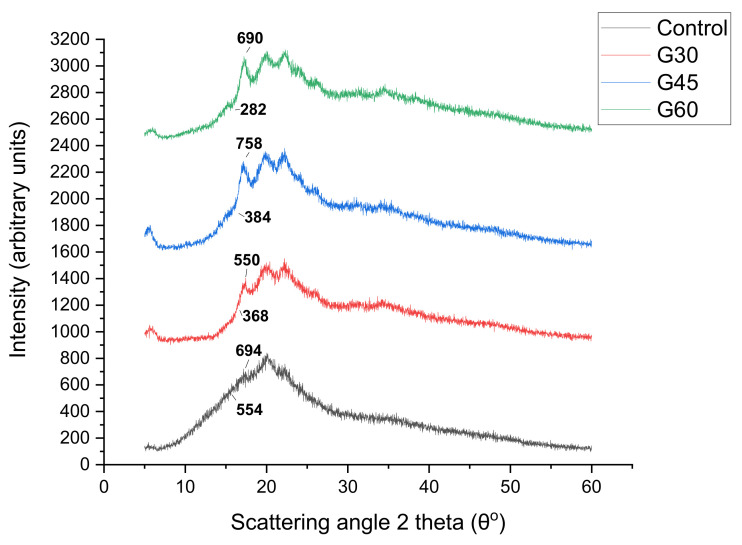
XRD of glycerol plasticized and unplasticized CS films.

**Figure 12:**
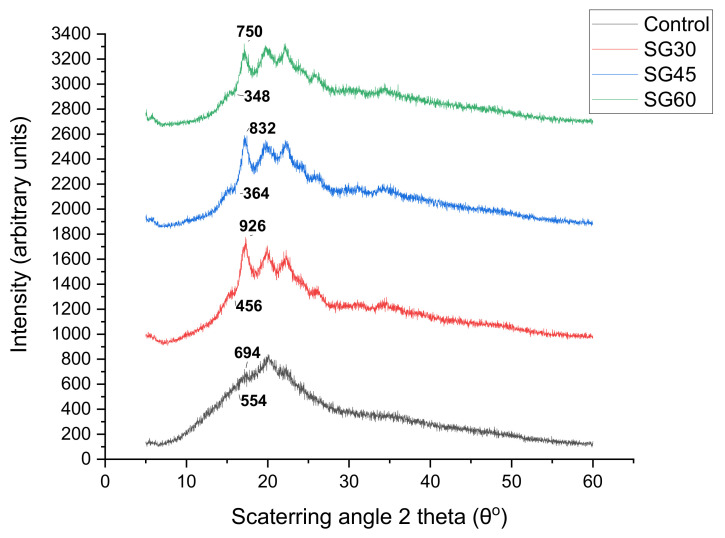
XRD of sorbitol/glycerol plasticized and unplasticized CS films.

The corresponding XRD patterns of the CS biopolymer films are shown in Figure 10, Figure 11 and Figure 12. The XRD analysis was conducted to determine the effect of plasticizer type and concentration on the crystal structure of CS-based biopolymer. The X-ray diffraction structures of CS-based films are introduced in Figure 10, Figure 11 and Figure 12. The results showed that the majority of plasticizer particles on CS films were gelatinized and retrograded, triggering a collapse of the crystalline starch system, thus forming an amorphous system. The CS-film retrograded has diffraction peaks at 15.14°, 17.4°, 18.6°, 20.11°, and 22.8°. These peaks were also consistent with the peaks presented by Paraginski et al. [64]. However, the addition on the crystalline structure of films of the plasticizer concentration regardless of the plasticizer type concerned. This was also mentioned by Sanyang, Ilyas et al. [65,66]. The X-ray diffraction patterns of CS-based films plasticized with S, G and SG at different concentrations (0%, 30%, 45%, and 60%) are presented in Figure 10, Figure 11 and Figure 12. A large amorphous region with crystalline peaks, as noted, was observed in the unplasticized CS film. CS films had crystallinity of 10° to 20° reflectance that was similar to the B-type diffraction pattern [67]. Similar remarks were made by Zhong et al. [68], who proposed the development of double-helical crystalline B- type with peaks at 2 θ to 17°.

The unplasticized CS films have X-ray diffraction patterns similar to those of glycerol (G- and SG-plasticized films) films, but the newly established top sank to 19° and G- and SG-plasticized films received the addition of glycerol. The peak of 19° corresponded to the crystalline V-type structure that demonstrated the presence of interactions between amylose-glycerol. García et al. [69] proposed that amylose favored heavy glycerol interaction. Gutiérrez, Pérez et al. [70,71] indicated that amylose within the starch was mainly responsible for starch-based film crystallinity. The V-type structure depicted in G- and SG-plasticizing films is due to the single helical structure created by amylose and glycerol interactions. In other words, the addition of glycerol to the starch solution disrupted their double helix concentrations by establishing stable V-conformation helices in a single chain. This contributed to the creation of glycerol amylose complexes [29,70,71,72]. In addition, the intensity of the peak diffraction increased as the concentration of glycerol increased from 0 to 60% for G and SG films. Zhong et al. [68] mentioned, the effect of glycerol on the properties of Kudzu starch films was studied and the crystallinity of films increased with the increase of glycerol concentration from 0% to 40%. Bergo et al. [73] also reported similar findings on the crystallinity index where properties increase from 0 to 45% using cassava starch films.

The S-plasticised films’ X-ray diffraction patterns showed a significant increase in peak intensity at 17.6°. The peak distinction became explicit, with the concentration of sorbitol increasing from 0 to 60%. The increase in the concentration of sorbitol (0–60%) has a major impact on the essential nature of CS films which is shown by its sharp, well-defined peaks combined with insignificant amorphous regions. On this basis, plasticized S30 films were extremely crystal clear, as opposed to plasticised S45 and S60 films. The broad diffraction pattern indicated less crystallinity. Additional sorbitol levels up to 60% increased the S60 film’s crystallinity as reflected in the presence of several sharp peaks. The high crystallinity of films S30 and S60 might be caused by the disorder in the intermolecular starch and hydrogen bonds between sorbitol and starch molecules and by their replacement during plasticization. Sanyang, Ilyas, Gutiérrez, Famá, Hu et al. [65,66,70,74,75] reported that an increase in crystallinity of starch films was strongly related to a decrease in film moisture content. Therefore, the increase in the crystallinity of S-plasticized films as observed Table 3 was associated with their low moisture content obtained in this study.

### 3.9. Tensile Properties

**Figure 13:**
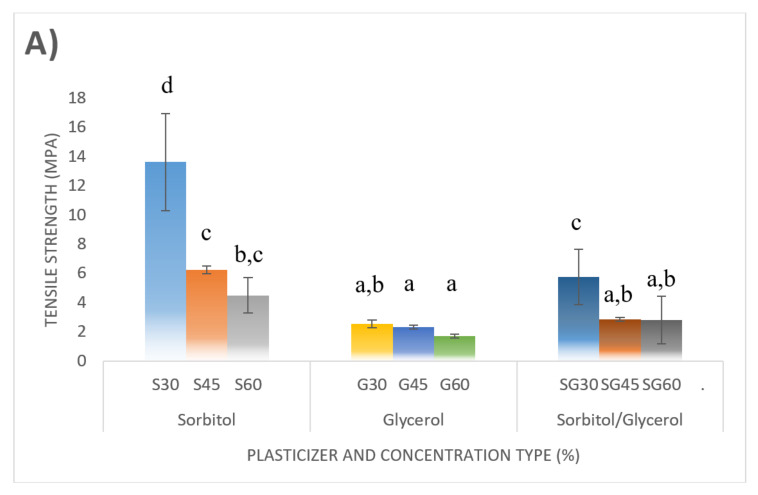

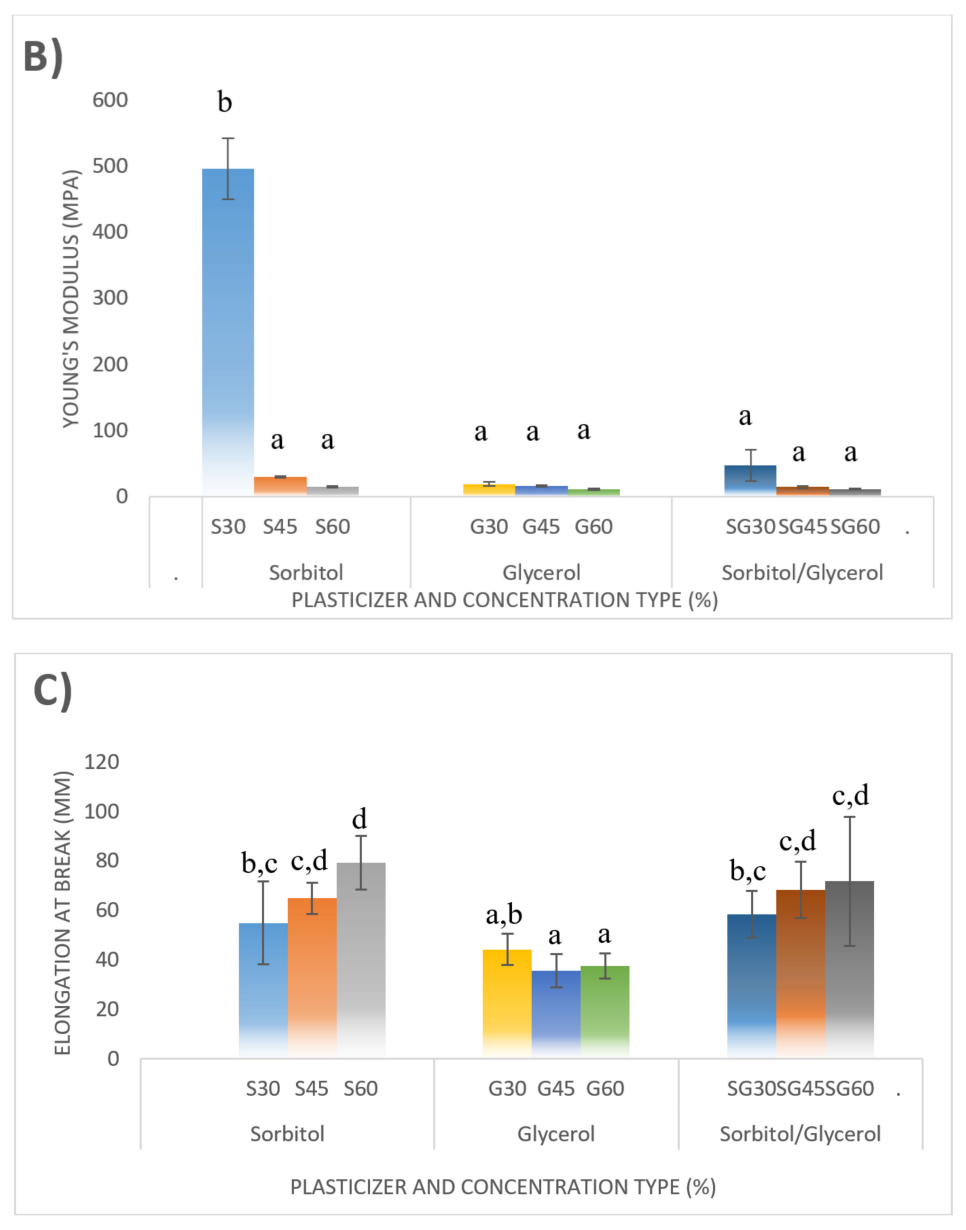
Tensile properties of corn starch biopolymer films. (**A**) tensile strength, (**B**) tensile modulus, and (**C**) elongation at break. Values with different letters (a–d) in the same column are significantly different (*p* < 0.05).

Figure 13 shows the effect of CS-based films with different plasticizer type and loading on mechanical performance. Tensile testing was conducted to determine the tensile strength (TS), tensile modulus (E), and break elongation (EB). This test was carried out to precisely determine the performance stress of the tensile and CS-base films of Young’s modulus, which was plasticized with different plasticizer types and concentrations. The results showed that the tensile strength of the tested films was reduced when the plasticizer concentration rose from 30% to 60%, irrespective of the plasticizer form. This was fully consistent with the preceding hypothesis [12,29]. The 30% sorbitol film recorded the highest tensile stress (13.62 MPa), which was higher than that recorded with 30% glycerol (2.53 MPa) and 30% sorbitol/glycerol (5.74 MPa). The expected understanding of high tensile stress at low plasticizer content was correlated with hydrogen bonds formed between starch and plasticizer molecules, where these bonds were strongly dominated by lower plasticizer content and reduced as the plasticizer content increased [36,41,48,51,53].

Thus, the tensile strength of S-plasticized films decreased from 13.62 to 4.48 MPa and that of G-plasticized films decreased from 2.53 to 1.7 MPa, as the percentage of plasticizers increased from 30% to 60%. Meanwhile, the lowest tensile stress values were reported in G-plasticized films, which corresponded to the same percentage range for plasticizers from 2.53 to 1.7 MPa. Many authors detected a decrease in the tensile strength of starch-based films due to increased plasticizer concentrations [36,41,48,51,53]. In the present study, the tensile strength for sorbitol films was greater than that observed by Ibrahim et al. [36], the investigators developed plasticized films by mixing cornstarch with 25% sorbitol and obtained 4.52 MPa strength. Likewise, the same authors added 55% sorbitol and obtained 3.04 MPa far from the current analysis. The tensile strength values of CS-sorbitol- plasticized films were generally higher than the values previously recorded for glycerol-plasticated corn films [76], cornstarch with stearic acid and glycerol [77], and cornstarch with xylitol and glycerol [78]. In this analysis, on the other hand, the tensile strength values for CS films were more than thymol and glycerol on corn starch [79], and plasticized glycerol and sorbitol sweet potato starch [80] in relation to elastic modulus (Young’s modulus) which determines the stiffness of materials. 

The higher elastic modulus value implies optimum stiffness. Figure 13B shows that the impact of plasticizer material (30–60%) on Young’s CS-plasticized film module had the same behaviour compared to their corresponding tensile stress. The rising plasticizer concentration from 30% to 60% resulted in a significant decrease of film stiffness: from 495.97 to 15.34 MPa for S-plasticized films, 19.43 to 11.83 MPa G-plasticized films, and 47.17 to 11.88 MPa for SG-plasticized films. This behaviour can be explained by plasticizers’ function in modifying starch network structure. By incorporating plasticizers into starch chains, they facilitated the formation of hydrogen bonds between molecules and weaken the solid intra-molecular attraction within the starch matrix. Young’s CS-plasticized film modulus was reduced and less rigid [36,41,48,51,53]. In summary, numerous parameters, such as the botanical origin of starch (Amylose/amylopectin ratio), the environmental conditions (temperature and humidity), and the process method and the plasticizers types and concentrations highly influenced the biopolymers’ mechanical role based on thermoplastic starch. These findings showed that sorbitol was regarded to higher efficiency in CS films than glycerol as the strongest plasticizer and the two combined. 

### 3.10. Scanning Electron Microscopy (SEM)

**Figure 14:**
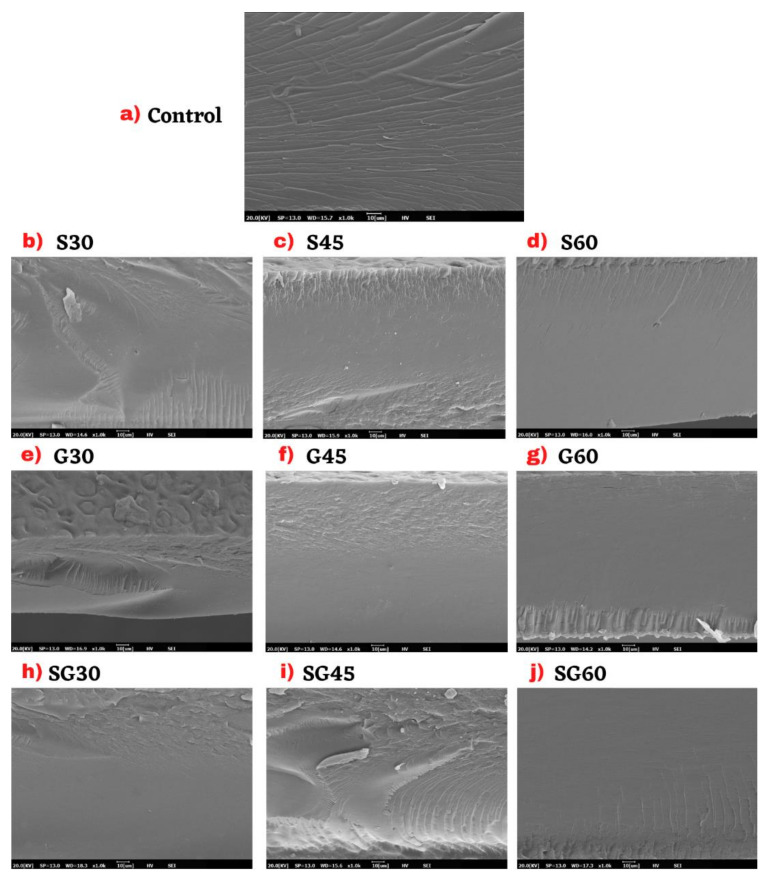
Scanning electron micrograph of CS films with various plasticizer and concentration.

SEM images of the fractured surfaces of CS film samples at the magnification of 1000× were shown in Figure 14. The film surfaces’ microstructures were examined to determine the comparison of surface morphology by plasticizer types and concentrations. The general aspect showed that the corn starch had consistently homogeneous surfaces and had a clear plasticizer cover. This was because the plasticizer functioned to building strong interaction bonds within the starch matrix, since the plasticizer and matrix were both carbohydrates of the same polarity [81]. The surface of the unplasticized CS film tended to have irregular break. The observed rough surface can be correlated with the remainder of the CS granules in the CS film polymer matrix. According to Ibrahim et al. [36], the SEM of native CS displayed relatively smooth and uniform surface, which reflected the morphological structure of CS. 

The films with 60% concentration of plasticizer presented a smooth surface without pores and no dissolved particles, as shown in Figure 14d,g,i, S60, G60, and SG60, respectively. This smooth surface was formed due to perfect and homogeneous stirring during film preparation. The introduction of the S30, S45, G45, and SG30 plasticizer in the pure starch film demonstrated such disturbances to film surfaces, due to the high temperature and continuous rattling, while the G30 and SG45 surfaces were coarse and filled with certain impurities and aggregated with none of the undissolved starch, during preparation and drying. Unlike the counterpart of the S-films which displayed better surface integrity than the G and SG film in all plasticizer concentrations, the films plasticized by 30 and 45% for G and SG plasticizers showed lower consistent surfaces with high porosity as well as microcracks.

However, appropriate additions to starch-based films of plasticizers help to overcome whole starch molecules. They enhance the structural stability and quality of the film surface [82]. Therefore, the highest concentration of plasticizers used in starch films production was 60% (*w*/*w* dry basis), the excess of this threshold was a thin incoherent film that was hard to peel off. By comparison, the prepared film containing less than 15% (*w*/*w* dry) plasticizer content seemed delicate, sticky, and difficult to remove from the casting plate [41]. Therefore, determining their properties became difficult. These findings were consistent with those described in previous literatures [36,48,83]. S-plasticized films proved to be relatively smoother, homogeneous, and compact for all different plasticizer concentrations in the current sample. These SEM images showed the successful interaction of sorbitol with CS molecules to weaken the starch’s intermolecular and intermolecular hydrogen bonds [41]. S-plasticized films’ observed surface features expressly justified their low moisture content and water absorption.

## 4. Conclusions

CS films were fragile without plasticizers with several visible cracks and were not easily removed from the casting surface. The introduction of plasticizer thus helped to inhibit the breakability and increase the versatility of CS films. Different variety and concentrations of plasticizers were used to analyze the physical and chemical effects of CS films. The results showed that the type and concentration of plasticizers affected the CS film thickness, density, and humidity. Gradually increasing the concentration of plasticizers from 30 and 60% reduced the density and water absorption properties of the films, nevertheless it also increased the film weight, thickness and humidity regardless of the plasticizer form concerned. However, the moisture and water absorption on S-plasticized films were least affected by the changing concentration of plasticizer. S-plastic films showed less moisture, solubility, and water absorption compared to G- and SG-plasticized films. Films with G-plasticizer, by comparison, showed lower film thickness and density than films with S-plasticizers. Overall, S-plasticized films exhibited the highest efficiency, in terms of physical and mechanical properties. The S30-plasticized films also demonstrated excellent mechanical properties for tensile stress and tensile modules with 13.62 MPa and 495.97 MPa, respectively. The effect on the solubility, thermal, electrical, soil burial, inflammability, and barrier properties of corn starch bioplymer of different plasticizers and concentrations should be done in future study to determine the best combination for the production of biodegradable packaging films and plastics.

## Figures and Tables

**Table 1:** General appearance of non-plasticized and plasticized CS films.

Label	Plasticizer	Plasticizer Concentration (%)	Appearance of Films
CS	-	0	Transparent, surface cracks, brittle and fragile, difficult to peel
S30	Sorbitol	30	Crystal clear, rigid, non-sticky, not brittle and not fragile, flexible, peelable
S45	Sorbitol	45	Crystal clear, rigid, non-sticky, not brittle and not fragile, flexible than S30, peelable
S60	Sorbitol	60	Crystal clear, rigid, non-sticky, not brittle and not fragile, flexible than S45, peelable
G30	Glycerol	30	More transparent, sticky, not brittle and not fragile, flexible, easy to peel
G45	Glycerol	45	More transparent, more sticky than G30 and SG30, not brittle and not fragile, flexible, easy to peel
G60	Glycerol	60	More transparent, more sticky than G45 and SG45, not brittle and not fragile, flexible, easy to peel
SG30	Sorbitol/Glycerol	30	Transparent, sticky, sturdy, rigid and not fragile, flexible, easy to peel
SG45	Sorbitol/Glycerol	45	Transparent, stickier than SG30, sturdy, rigid and not fragile, more flexible compared to G30, easy to peel
SG60	Sorbitol/Glycerol	60	Transparent, stickier than SG30, sturdy, rigid and not fragile, more flexible compared to G30, easy to peel

**Table 2:** Physical properties of corn starch films incorporated with various plasticizer types and concentration.

Plasticizer and Concentration	Thickness (mm)	Weight (mg)	Density (g/cm^3^)	Moisture Content (%)
Control	0.10 ± 0.01^a^	0.04 ± 0.02 ^a^	1.65 ± 0.02 ^f^	11.64 ± 0.1 ^a,b^
S30%	0.16 ± 0.02 ^a,b^	0.07 ± 0.02 ^b^	1.45 ± 0.05 ^e^	9.25 ± 2 ^a^
S45%	0.17 ± 0.02 ^a,b^	0.08 ± 0.02 ^b^	1.44 ± 0.02 ^e^	10.04 ± 2 ^a^
S60%	0.22 ± 0.03 ^c^	0.09 ± 0.02 ^b^	1.43 ± 0.02 ^c,d^	9.28 ± 2 ^a^
G30%	0.14 ± 0.02 ^a,b^	0.06 ± 0.02 ^a,b^	1.39 ± 0.01 ^c,d^	14.7 ± 2 ^b,c^
G45%	0.16 ± 0.02 ^a^	0.06 ± 0.02 ^a^	1.33 ± 0.02 ^b^	17.27 ± 2 ^c^
G60%	0.19 ± 0.03 ^b,c^	0.07 ± 0.02	1.30 ± 0.02 ^a^	16.55 ± 2 ^c^
SG30%	0.18 ± 0.02 ^b,c^	0.07 ± 0.01 ^b^	1.40 ± 0.01 ^d^	9.11 ± 2 ^a^
SG45%	0.19 ± 0.02 ^b,c^	0.08 ± 0.01 ^b^	1.39 ± 0.01 ^c,d^	12.56 ± 2 ^a,b^
SG60%	0.20 ± 0.02 ^b,c^	0.09 ± 0.02 ^b^	1.38 ± 0.02 ^c^	14.99 ± 2 ^b,c^

Values with different letters (a–f) in the same column are significantly different (*p* < 0.05).

**Table 3:** Crystallinity index of CS films.

Film Sample	Crystallinity Index (%)
Control	20.17
S30	39.86
S45	44.27
S60	54.87
G30	33.09
G45	49.34
G60	59.13
SG30	50.75
SG45	56.25
SG60	53.60

## Data Availability

Not applicable.
